# Corrigendum to “Genome-Wide Identification of Long Noncoding RNAs in Human Intervertebral Disc Degeneration by RNA Sequencing”

**DOI:** 10.1155/2019/3132626

**Published:** 2019-09-26

**Authors:** Bo Zhao, Minjuan Lu, Dong Wang, Haopeng Li, Xijing He

**Affiliations:** ^1^Department of Orthopaedic Surgery, The Second Affiliated Hospital of Medical College, Xi'an Jiaotong University, Xi'an 710004, China; ^2^Department of Respiratory Medicine, Baoji People's Hospital, Baoji 721000, China

In the article titled “Genome-Wide Identification of Long Noncoding RNAs in Human Intervertebral Disc Degeneration by RNA Sequencing” [1], there was a missing grant number. Thus, the Acknowledgments section should be updated as follows:

This study was supported by the grants from the National Health and Family Planning Commission (Grant no. 2011-1) and ShaanXi Natural Foundation (Grant no. 2016JM 8136).
